# “Not just dogs, but rabid dogs”: tensions and conflicts amongst research volunteers in Malawi

**DOI:** 10.1080/11287462.2018.1509925

**Published:** 2018-09-03

**Authors:** Mackwellings Phiri, Kate Gooding, Deborah Nyirenda, Rodrick Sambakunsi, Moses Kelly Kumwenda, Nicola Desmond

**Affiliations:** a Malawi Liverpool Welcome Trust Clinical Research Programme, Blantyre, Malawi; b Liverpool School of Tropical Medicine, Pembroke Place, Liverpool, UK

**Keywords:** Trust, community representative, community counsellor, community engagement, HIV self-testing

## Abstract

Building trust between researchers and communities involved in research is one goal of community engagement. This paper examines the implications of community engagement for trust within communities, including trust among community volunteers who assist with research and between these volunteers and other community members. We describe the experiences of two groups of community volunteers recruited as part of an HIV and TB intervention trial in Malawi: cluster representatives, recruited both to act as key informants for TB suspects and mortality reporting and to identify and report community concerns, and community counsellors, recruited to provide semi-supervised HIV self-testing. We examine tensions experienced due to playing multiple roles, and the implications of volunteer responsibilities for short- and long-term community relationships. Data was collected through a workshop, in-depth interviews and focus group discussions with volunteers and community members. While the volunteer system initially enhanced trust among volunteers and with the community, relationships deteriorated when cluster representatives assumed an additional supervisory role part-way through the trial. Combined with challenging recruitment targets and unequal power relations between volunteers, this new role damaged trust, with implications for volunteer well-being and social relationships. These experiences suggest researchers should consider potential social implications when designing community engagement systems.

## Introduction

Community engagement (CE) is increasingly seen as important in biomedical research. There is no universal understanding of the terms community or community engagement. For instance, a community may refer to a group of people residing within a specific geographical location, a group with similar characteristics, or a group served by one health facility (Gbadegesin & Wendler, 2006; Marsh, Kamuya, Rowa, Gikonyo, & Molyneux, 2008; Tindana et al., 2007). We define community as a group of people living within a specified geographical location, and CE as the practice of relevant partners working together to deliver shared goals and interests (Tindana et al., 2007).

The attention to community engagement is based largely on ethical goals (Simon & Mosavel, 2010). These goals include ensuring safety of research subjects through understanding community views on the research and identifying potential risks and means of averting those dangers (Anderson & Solomon, 2013; Dickert & Sugarman, 2005), ensuring that study procedures meet community needs (Boga et al., 2011), and protecting communities from exploitation, particularly in developing country settings (Chantler et al., 2013; Gbadegesin & Wendler, 2006). Community participation in research decision-making can help to ensure that research responds to community needs, and ultimately that research benefits are shared equitably between researchers and participating communities (Pratt et al., 2013; Wallerstein & Duran, 2010). Supporting these aims, development of trust between communities and researchers is often understood as a key function of engagement (Anderson & Solomon, 2013; Dunn, 2011; Kamuya, Marsh, Kombe, Geissler, & Molyneux, 2013; Molyneux, Peshu, & Marsh, 2005).

One approach to community engagement is the use of community volunteers as an interface between researchers and communities (Marsh et al., 2008). Terminologies for community members engaged in research vary, and include community health workers, community health helpers, village reporters (Chantler et al., 2013), community volunteers (Sambakunsi, Kumwenda, Choko, Corbett, & Desmond, 2015), or key informants (Jahn et al., 2007). The way that community volunteers are engaged in research takes different forms, for example, it may be as individuals or as members of community advisory groups or boards. The roles undertaken by community volunteers also vary. For example, they may be asked to act as representatives who share feedback from communities (Clodagh, 2015), a role more closely aligned to the ethical goals discussed above, or to play roles focused on undertaking or facilitating research, such as helping to recruit participants (Simon & Mosavel, 2010) or collect research data (Jahn et al., 2007)

In such diverse roles, community volunteers, especially those working as recruiters or collecting data, may experience challenges, including tensions in meeting the needs of the research study whilst continuing to serve their communities. They may face incompatible requirements of meeting performance expectations while adhering to ethical practice (Kamuya, Theobald, et al., 2013), for example, ensuring high participation rates while respecting voluntary participation (Sambakunsi et al., 2015). Managing personal obligations alongside research needs can also create pressures for community volunteers and limit their ability to work effectively (Sambakunsi et al., 2015). These and other pressures can lead to loss of resources and energy for community volunteers engaged in research, and cause distress and demotivation (Attree et al., 2011).

Fieldworkers, who have more formal contracts with research institutions than research volunteers (Molyneux et al., 2013), may play similar roles and face similar challenges to those described for volunteers. A key issue highlighted in the literature on fieldworkers is the role of trust, both in enabling their work and as an outcome for their activities. For example, it is easier for fieldworkers to obtain consent if community members have confidence in them (Kamuya, Theobald, et al., 2013). However, closeness of fieldworkers to the community may also hinder trust in the fieldworkers (Chantler et al., 2013), because participants might be concerned about privacy and confidentiality (Molyneux et al., 2013). Furthermore, fieldworkers may also find it hard to manage pressure linked to set recruitment targets and to maintain respectful relationships with communities, and are sometimes compelled to adopt seditious practices such as data forgery in order to meet performance expectations (Brown, Long, Weitz, & Milliken, 2001; Kingori & Gerrets, 2016).

While there is growing discussion about the perspectives of community volunteers in developing countries, there is limited research focused specifically on their views about the inherent tensions around their roles. There is also limited evidence on the unintended and longer-term consequences of engagement initiatives for the engaged persons (Attree et al., 2011), including implications for trust.

Trust is an interpersonal concept, centred between people, people and organizations, or people and events (Chantler et al., 2013). Trust can be voluntary or involuntary (Gilson, 2003). Voluntary trust is based on expectations of how others will behave in relation to yourself in the future. These expectations may be disappointed and, if so, will generate negative outcomes. Forms of conduct that usually underlie voluntary trust include being competent, open and dependable. Involuntary trust may exist in relationships that result from lack of choice or occur in the context of inequality, for example, relationships between health care providers and patients may appear as a form of dependency (Gilson, 2003). For the purpose of this article, we focus on voluntary trust between individuals embedded within communities.

This paper describes the experiences of two groups of community volunteers recruited to work within their own communities participating within an HIV/TB intervention trial in Malawi: cluster representatives (CRs) recruited as key informants for TB outcomes and mortality and concurrently as representing and reporting community concerns, and community counsellors (CCs) recruited more formally to provide semi-supervised HIV self-testing. We examine the tensions experienced as a result of being asked to play multiple roles, and the implications of their volunteer responsibilities for short- and long-term community relationships and trust.

## Research methods

### Study setting: the HitTB intervention as a case study

The study presented here took place between March 2015 and December 2015, in urban Blantyre, Malawi. It emerged out of a community-based intervention trial that ran from 2012 to 2015 investigating whether community-level active TB case finding and intensified TB prevention through regular semi-supervised, HIV self-testing (HST) and access to treatment could reduce TB incidence (Choko et al., 2015; Kumwenda et al., 2014; Kumwenda et al., 2018; Sambakunsi et al., 2015). This trial was implemented in 28 clusters (14 intervention and 14 control) of high density and low-resource urban settlements with relatively high rates of social migration and many residents reliant on a range of informal and unreliable income-generating activities. The HST study was implemented in the intervention clusters only, whereas all clusters received the standard of care which included active TB case-finding and extended routine monitoring and evaluation, enumeration of households and survey to document if participants know their HIV status and whether they are receiving care and support for increased Isoniazid Preventive Therapy through routine HIV care services during the course of the trial.

Map showing intervention and control clusters within high density and low-resource urban communities in Blantyre, Malawi.


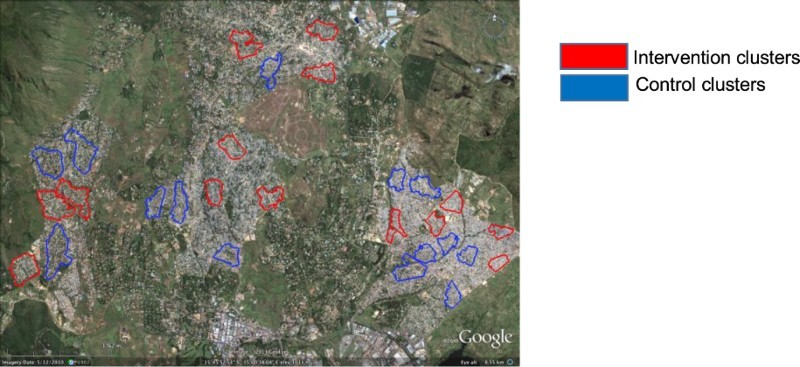


To support the trial, a community liaison system was established as a key component of an integrated community engagement strategy, which aimed at ensuring there was regular and sustained dialogue between the community and the research team. A total of 112 volunteers (56 men and 56 women) were recruited as cluster representatives (CRs), four within each intervention and control cluster. The HST intervention was driven by another set of volunteers in intervention clusters only; community counsellors (CCs), 28 in total, two representing each intervention cluster (14 men and 14 women). So, half the number of CRs worked in the same intervention clusters as CCs.

### Community-driven selection of CCs and CRs

CCs and CRs were identified and selected concurrently through an elective process led by the community. The selection took place during community sensitization meetings organized by the research team. Before selection, the research team provided community members with basic nomination criteria, which specified that a CC/CR be: willing to volunteer, a regular cluster resident (defined as sleeping in the area for more than a fortnight in every month), honest and able to maintain confidentiality. A counsellor was additionally required to have at least a Junior Secondary Certificate (GCSE equivalent) since they would be required to attend national HIV Testing and Counselling (HTC) training in order to deliver semi-supervised HST. When individuals had been put forward and votes taken, local leaders and other stakeholders counted the votes and consolidated results publicly.

### Official functions of CCs and CRs

CCs were entrusted with the delivery of home-based HST services. Initially based at their own home with clients coming to them but eventually moving door-to-door to meet HST targets (Sambakunsi et al., 2015), they distributed HST test kits (OraQuick ADVANCE HIV I/II), counselled clients before and after HST, screened for TB among self-testing clients, and referred clients for appropriate health services. In contrast to the CCs, CRs had a number of roles: they were responsible for providing community feedback on the intervention, such as fears and concerns related to participation and conduct of the trial. They also helped with community sensitization through community meetings and door-to-door distribution of study brochures. Further, CRs collected and reported information on mortality and potential cases of TB occurring in their cluster. If someone had prolonged cough for example, CRs reported the individual to CCs for TB screening; and if someone died, they reported to study nurses who then attended to conduct a verbal autopsy. This was part of a key informant system designed to monitor mortality and TB incidence both in the intervention and control clusters. This gave CRs two potentially conflicting roles; one as key informants for the research team and the other of representing the interests and concerns of the community.

Though CCs and CRs served on an officially voluntary basis, they received some monetary benefits for their work, offered as a token of appreciation for their time but disparately apportioned: CCs received MWK 14,000 (*approx. 56 USD in 2012*) whereas CRs received |MWK 2,000 (*approx. 11 USD*) monthly.

### Additional duties for CRs following changes in procedures for recruitment by CCs

CRs took on additional roles when the research team introduced recruitment targets for CCs. The intervention aimed to reach at least 80% HIV self-test uptake amongst adults aged 16 and above, resident in intervention clusters within a two-year timeframe. However, after one year of the study, the research team realized that uptake figures were lower than anticipated. Instead of 10 clients a week, CCs were on average recruiting 4 clients. This was largely due to a passive recruitment approach reliant on HST clients self-presenting at CCs homes. The community engagement team then held consultations with the principal investigator, field supervisors and CC representatives to identify strategies for improving recruitment through a door-to-door testing approach. Following these consultations, and given indications that CCs were deliberately delaying recruitment because of unhappiness with the amount of compensation they were getting, the research team decided to reimburse CCs based on performance. If a CC reached 100 clients in a month for example, they would receive an extra MWK40, 000 (*approx. 240 USD in 2012)*.

While the expectation was that improved compensation for CCs would increase testing uptake rates, the research team also wanted CCs to remain adherent to the study protocol when recruiting, and thus entrusted CRs with another role as watchdogs for quality assurance. To support this role and as a result of suspicions of false reporting by CCs, study nurses provided CRs with names of clients who had been reported to have self-tested, selected randomly from enrolment registers to check eligibility: residency within the designated intervention clusters and aged 16 years or above. The research team informed CCs about quality assurance. However, CCs and communities were not informed that CRs were to play this role, and CRs were instructed not to share with CCs whatever they found. The possibility of this arrangement causing tensions and distrust between CCs and CRs was not foreseen at the time.

As a result of suspicions of developing conflict within the community-based intervention teams, we explored the experiences of the CRs and their multiple and often conflicting roles as key informants, watchdogs and representatives of the community, interrogating the concept of simultaneous representation of two institutions; the community and the research institution and the impact of this on their relationship in both the short and long term with two different social groups: community peers and CCs.

## Data collection

Data collection began with a one-day participatory workshop (PW), conducted to solicit broad ideas about how community representatives understood and experienced their dual role as key informants and representatives of the community. MP (research assistant) and RS (senior community liaison officer at the time) co-facilitated the workshop, designed in such a way that issues could be explored freely and in the participants’ own terms. MP previously worked on a qualitative longitudinal study that had recruited participants from HitTB and was familiar with the intervention trial. During this time, he had frequently interacted with the study communities during interviews and focus group discussions, and particularly with both CCs and CRs, who had always helped him with the recruitment of study participants. The rapport previously built between MP and the communities prior to his role in this study proved invaluable since participants felt free to discuss their experiences of the intervention trial openly with him.

Seventeen CRs (9 men and 8 women) participated in the workshop, recruited from across intervention and control clusters. They were conveniently selected based on availability and willingness to attend, rather than purposively according to specific predefined criteria. The workshop involved three sessions. First, participants sat in three smaller groups to discuss their roles and experiences, each group recording their ideas on a flipchart. Second, using the flipchart notes, each group presented their discussions. Third, the three groups merged for a single group discussion in which the two facilitators examined in-depth the issues that groups had separately raised. Each session lasted approximately 1 hour.

We conducted further focus groups and interviews to complement findings of the PW. We conducted five focus groups: one with cluster residents, one with community counsellors, and three with cluster representatives. There were 8-12 participants per focus group, and the discussions lasted on average two hours. Because the topics being investigated were not gender sensitive, we combined men and women. We also conducted seven interviews with a sample of key stakeholders: one with a female cluster resident, three with CCs (one man and two women), and two with CRs (one man and one woman). The interviews lasted on average 30 minutes. Some of the workshop participants were purposively sampled for the focus groups and interviews in order to follow up on issues reported from specific clusters. All interviews, focus groups and workshop discussions were conducted in Chichewa, the main local language within the study context, and audio recorded after obtaining written consent (Table 1).

**Table 1.** Summary of study participants.

Participant type	Gender					
	Male			Female		
	IDI	FGD	PW	IDI	FGD	PW
CRs	1	15	9	1	10	8
CCs	1	6		2	6	
Community members		4		1	4	

## Data analysis

Data analysis and data collection ran concurrently and iteratively so that emerging findings could be followed up through later data collection. Flipchart notes from the workshop were typed up and saved as data scripts. Audio recordings were transcribed verbatim in Chichewa, and all data scripts were imported into NVIVO 10 software (QSR, Melbourne, Australia) for better organization and analysis. MP and RS independently read the same four Chichewa transcripts and coded them inductively. Each author came up with a coding frame. The two separate coding frames were compared and discussed, and then merged to generate a single coding structure. After comparing the two coding frames, some of the codes were maintained and others were merged. The coded data were analysed using a thematic approach to identify key themes emerging from the data that were then discussed with co-authors. Representative quotes were extracted to represent each of these themes and translated into English.

## Study authorization

Permission to conduct this study was obtained from The Malawi College of Medicine and The Liverpool School of Tropical Medicine research ethics committees.

## Results

Community misconceptions about the trial influenced their attitude towards community volunteers working for the trial (i.e. CRs and CCs). Alterations made to trial procedures to boost recruitment and safeguard data quality damaged the relationship between CCs and CRs and increased pressure for the CCs to perform. Data quality checks performed by CRs on self-tested clients diluted community trust in the CCs because of a perceived breach of confidentiality that CCs had initially assured clients during an informed consenting process. We discuss these findings in detail in this section.

## Initial distrust in the study concept transitions to trust through the roles of CRs and CCs

Relations between CCs and CRs and the community they served changed over time, and oscillated between trust and distrust across each group. At the outset, the community reacted to the intervention with distrust due to unfamiliarity with HIV self-testing, an approach that residents viewed as peculiar and risky.

*HIV self-testing was new. People had been used to finger pricking and were questioning: “Oral test kits, what kind of test kits are these? We’ve never seen this before.” Today, if something new is introduced, you are going to hear, “This is satanic, they want to collect blood from you, that thing will suck blood from you.”* [FGD, female community member]

This failure to comprehend how the procedure worked influenced ideas about the study being satanic, and resulted in the CRs being viewed with suspicion and rejected.

*People did not welcome us in their homes if we wanted to talk to them about the study. Some chased us away: “Go back! I don't want to see you around my house! You might invite to my home the devils you worship in your organization!” Some would send their dogs after us.* [PW, male CR]

Proximity to the community was another reason why people formerly did not trust CCs and CRs. Residents feared that their privacy regarding HIV status might be publicly disclosed and were hesitant to approach the volunteers, as one community member explains:

* … we’re worried about confidentiality because they [CC] were coming from the same area as us. We would ask them, “How can we prove that you are going to protect our privacy?” … if you were going out, you were thinking that you might hear people talk about what you discussed with them, but a month went by without hearing anything from anyone. That's when we proved that they were confidential.* [IDI, female community member]

However, through the community mobilization that CRs performed, people got more informed about the study and assured about safety of their privacy, and community distrust in the CCs reversed:

* … if they gave you the paper [study brochure] to read, they would also tell you: “our colleagues will also stop by with oral test kits, everything will be confidential, and, if your test result is out, it's your choice whether you want to tell them or not.”* [FGD, male community member]

Testimonies of demonstrated ability to safeguard clients’ privacy from those already self-tested also contributed to increasing community approval of the CRs:

*We were handing out flyers and a woman asked her landlord: “Can I talk to these people about my issue? Can I trust them with my privacy?” The landlord said yes, and then she let us into her house and told us about her marriage problems including information about being initiated on HIV/AIDS treatment.* [FGD, male CR]

Community confidence in the volunteers was further negotiated through the role of local authorities. Village chiefs often requested their communities to welcome and listen to the researchers. As the subsequent quote illustrates, this indicated project authorization by the chiefs, and in turn increased trust and acceptability of the role of the CCs and CRs:

*Chiefs helped the study a lot because they informed people about what was happening in their areas. They announced at funerals: “People will be visiting you, they are from HitTB. Help them with the information they need because they are conducting a study.” When people heard this, they knew that the chief had allowed us to work in the area, and you would easily be accepted when you visited them.* [FGD, male CR]

## Changes in study procedures amplify teamworking relations between CCs and CRs

From the outset community-based CCs and CRs worked together as a unified team, mutually supporting each other's roles. Initial passive self-presentation for HST meant that CCs delivered the service directly from their homes and individuals were able to collect the kit and take it away with them. However, this initial approach faced challenges since firstly it relied on individuals taking the initiative to self-present and secondly, even when willing, residents often had problems locating where the CCs lived and thus in collecting the kit. This led to a change in approach with the encouragement of door-to-door sensitization to encourage residents to visit CCs, and required an additional role for the CRs to perform. If someone wanted HIV self-testing, CRs would escort them to or simply show them where the CCs lived. In return, CCs adopted an increased role in supporting the key informant component of CR activities, passing on information to CRs following any deaths occurring within the cluster in which they were operating. This reciprocity developed into a mutual reliance between CCs and CRs and fostered trusting partnerships, as one CC explains:

*We considered CRs as colleagues. If they identified a TB case suspect, or came across someone critically ill, they passed the information on to us.* [FGD, male CC]

## Disruption of trust between CRs and CCs through recruitment targets and watchdog roles

As a result of insufficient coverage of HIV self-testing and the study requirement to achieve 80% uptake across each cluster a system of targets was introduced after a year of the intervention with incentivization for CCs. In order to facilitate the meeting of these targets and particularly to ensure reporting of numbers tested was accurate and reliable (those testing were actual cluster residents and were above 16 years of age), CRs were requested by the research staff to take on a further role. They became “watchdogs” over the CCs, colleagues with whom they had previously worked hand in hand and naturally tensions emerged and previous mutual support between CCs and CRs was dramatically reduced and in some cases stopped altogether.

*Cooperation stopped when CRs were given the responsibility of checking if study participants self-tested … *[IDI, male CR]

* … especially when they had introduced Quality Assurance and used cluster representatives to enforce it, the enmity that was there [between us and CRs] was as if we were not working for the same organization.* [FGD, male CC]

For CCs the recruitment incentive linked to targets was significant in the context of daily livelihood challenges and a desire to increase income, whilst working within the study. On the one hand, it encouraged the CCs to work harder and spend more time recruiting.

*Targets made us work harder so that we can be rewarded. The allowance plus your usual monthly compensation meant that your income was improved.* [FGD, female CC]

On the other, the targets exerted immense pressure on the CCs as documented elsewhere (Sambakunsi et al., 2015), and created an incentive for the CCs to fabricate results such as the example below of a client describing how a CC made her self-test several times in order to meet a target:

* … he invited me to his house and told me to mention names of all my relatives, dead and alive, using fake dates … he said they had given him a target to meet within 3 or so days … he gave me test kits enough for the [ten] people I had mentioned and I used them on myself … he said he would give me money.* [IDI, female community member]

The CR watchdog role was meant to minimize such behaviours amongst CCs. In some cases, this monitoring role led to problems for the CCs. One CC was dismissed on account of making up results, and both the community and CCs blamed CRs for this dismissal:

*Some of the counsellors cheated and hated us for reporting them. Most of us are hated even today. We don't see each other eye to eye. They still blame us because one of them got sacked.* [IDI, male CR]

Over time and as the increase in incentives extended the pay differential between CCs and CRs, resentment built and increased tensions between those performing each role, exacerbating growing social discord within study clusters which has endured beyond the completion of the study. CCs looked upon themselves as superior to CRs due to higher compensation and the HTC training they received and, combined with the “watchdog” role of the CRs over their work ethic they often suspected CRs of wanting to steal their CC position.

*Counsellors were above us and reporting them seemed like you were interested in the counsellor position.* [FGD, male CR]

*The [HTC] training gave us job skills and they [CRs] were so bitter about it. They made false reports to the office, so that counsellors should look incompetent and not be trusted, because they were after the same position.* [FGD, male CC]

Though compensation and HTC training seemingly placed CCs above CRs, the watchdog role implicitly gave the latter more social power over the former, especially since CRs’ reports on CCs’ performance now determined whether CCs remained in their role. Moreover, the research team had instructed CRs not to share their findings with CCs, increasing suspicions of underlying subterfuge within communities. This disrupted the previously successful, existing social norm of mutual support between CCs and CRs and, as the quotes below illustrate, created mutual animosity:

*Our relationship with counsellors was like that of a cat and a rat … we were told not to disclose to the counsellors if we found that someone was recruited from outside the cluster.* [FGD, male CR]

*They put in a dog [CR] ready to bite … *[FGD, male CC] … *and it was not just an ordinary dog, but a rabid one.* [FGD, female CC]

These ideas of CRs as chasing CCs and ferociously reporting on CCs’ work show how this system eroded trust and friendship; CCs’ description of CRs as rabid dogs reflects their feeling that CRs were viciously seeking to ruin their relationship with the researchers. CCs’ distrust in CRs emerged largely in the context of CRs’ covert reporting to the researchers, behaviour that was in complete contrast to the former collaborative and open team working relationships. This led to fear amongst CCs that they might be reported by CRs as having cheated.

The tensions that arose in relation to the redefined roles surfaced in all study sites, and were particularly a recurrent theme in the participatory workshop, where CRs universally discussed issues of distrust between them and CCs, as one male CR said, “There was so much distrust between [cluster] representatives and [community] counsellors. They [CCs] felt we were intruding and looking to discredit their work … ” The majority of the CRs that attended the workshop were from the same clusters as CCs, and their reports of a difficult relationship with CCs in their respective clusters were identical. This perhaps confirms that conflicts between CCs and CRs were common to the clusters.

## Communities lose trust in CRs and CCs due to their multiple roles reigniting concerns around confidentiality

Multiple allegiances were the primary reason for a shift in community perceptions towards CRs over time. In the event of death requiring verbal autopsy for example, CRs, as key informants charged with reporting the death, located the bereaved household for study nurses to conduct autopsies. Consequently, whilst they had been considered as representatives of the community and routes to having their concerns heard, if a death occurred, CRs would be seen escorting members of the research team, and their previous role as community support would be replaced by the perception that they were on the side of the researchers representing their interests rather than those of the community. This led to a decrease in trust previously established between CRs and community members.

*When we had just the CR role, people knew we were on their side, and that we would stand up for them if anything bad happened concerning the study. They knew we weren't employed … but when we were given the [key informant] role, we appeared to have been more on the side of the researchers. That's when they stopped trusting us.* [IDI, male CR]

Further, the growing tensions previously described between CCs and CRs also had consequential effects on relationships between CRs and community members. For example, in the case where one CC had been sacked on data falsification grounds due to the CR having reported malpractice, community members criticized CRs as spying on the CCs. These guarded perceptions persisted even after the trial finished, demonstrating a longer-term impact of study dynamics, as illustrated in the quote below:

* … checking counsellors’ work made the community doubt us. Even today some of the people we used to spend time with now avoid us, suspecting that we’re going to report what they say. They see us as journalists, coming to nose around. One household said to us, “You still want to betray your friends? You want to report your friends so that they get sacked?”* [IDI, male CR]

Community distrust in CCs re-emerged because of a perceived breach of secrecy linked to the watchdog role that CRs performed. When recruitment quotas were set and CRs were dispatched to check eligibility of clients, cluster residents were concerned that their privacy had been disclosed, and suspected CCs of sharing details of the clients with CRs, as demonstrated below:

*Clients would ask you: “Somebody [CR] came to my house asking about my test result. You said my test result was confidential, so how come he [CR] wants to know the outcome of my testing?”* [IDI, female CC]

Despite this periodic disbelief in the CRs amongst the communities they represented, and some longer-term distrust as a result, that lasted beyond the study, there were also many demonstrations of positive trust sustained after study completion. Many community members continued to ask CRs for advice on health even when the study had closed, demonstrating a positive short-term residual impact of CR activities. When discussing post-trial roles, one of the things that CRs emphasized was the experience of frequently referring people to the hospital, and their experiences indicated some degree of community members depending on them for linkage into health services. This was reported in all study sites.

*There was death and members of the family came to us: “we are worried that our relative may have died of TB and that we might have been infected.” When I asked them to go to the hospital, they said, “Give us a referral letter so that they can attend to us quickly.”* [IDI, female CR]

*People got used to coming to us for advice; if they had TB or HIV, they would come to us for guidance. Even today they are still coming to us for advice. This time we ask them to go to the hospital because the study closed.* [IDI, male CR]

## Discussion

Our findings show how the use of community volunteers has the potential for both positive and negative impacts during and beyond the end of community-based research, creating both elements of trust and distrust between different project related roles and community members. In particular, we show how different, often conflicting roles and research driven requirements such as recruitment targets can lead to tensions, and that these tensions may impact on social relations during and after the completion of research studies. Specifically, we focus on community representatives recruited as key informants for TB outcomes and mortality and concurrently as representing and reporting community concerns and community counsellors recruited to provide semi-supervised HIV self-testing within the context of a community-based HIV/TB intervention trial, HitTB, in urban Blantyre, Malawi.

We show that whilst those who design community-based research may have the best of intentions, they are often blind to the implications of recruiting community-based volunteers, especially if these volunteers have differential and hierarchical roles within the study. Whilst we define these roles as community volunteers, their actual work reflects that of fieldworkers, rather than volunteers, since they are in receipt of salaries, and their actual responsibilities are driven by the needs of the study, rather than those of the community. It is perhaps this conflict between their responsibilities and the definition of their roles that creates the tensions that undermine the many positive aspects of employing recruits from the communities targeted in community-based interventions. Similarly to Reynolds et al. (Reynolds, Cousins, Newell, & Imrie, 2013) in work exploring the impact on social relations of the proximity of trial staff to community members affecting HIV surveillance in South Africa, we emphasize that it is imperative to understand these potential implications when designing interventions that rely on and in turn, influence for the future, social relations between communities participating in the research and those charged with delivering it. Communities need to be understood not as homogenous and harmonious, but as diverse groupings with ongoing inequalities and power relationships (Enria et al., 2016) and community-based research should understand these underlying inequalities and power differentials and their potential impact on them.

Some of our findings reflect research findings reported elsewhere regarding community volunteers and fieldworkers engaged by research. Other studies have found that pressure to manage recruitment creates challenges for the frontline staff responsible for recruiting. For example, in circumstances where resources necessary for supporting recruitment are limited recruiters face extra burdens in meeting research goals (Brown et al., 2001). Similarly to our findings, other studies have shown that multiple roles of community volunteers as representing community interests and advancing goals of the study may lead to tensions in fulfilling these dual roles (Molyneux et al., 2013). Concerns about breaches of privacy and confidentiality have also been identified where community members are close to or have prior relationships with prospective participants (Molyneux et al., 2013; Shedlin, Decena, Mangadu, & Martinez, 2011; Simon & Mosavel, 2010).

The need for multiple channels of engagement through the respective roles of CCs and CRs was clearly defined within the context of the intervention design as community-based. It was also clear to the research team, driven as they were by the need to reach targets for HST uptake in order to sufficiently power the results but whilst the former was described in the initial recruitment process for both positions, in contrast, the latter as the underlying cause of the changed role of CRs to monitors for CCs was not communicated either to the CCs or the community. It was clear from the impact on subsequent relationships, some of which extended, as we have noted, beyond the duration of the trial itself, that had this been communicated the community-level and longer-term impact might have been mitigated. Balancing research-driven requirements and ethical requirements in the context of such community-based research continues to be ridden by tension and conflict and this paper describes some of these (those of social relationships and trust) clearly. Had greater attention been paid to social relationships in the communities targeted by the intervention, trust might have been maintained.

Trust is important for health systems and development because it underpins cooperation throughout the system that is essential for health production and society building (Gilson, 2003). Current literature on trust related to community-based research tends to focus on how community engagement increases trust in research (Molyneux et al., 2005; Quinn, Kass, & Thomas, 2013), trust in research institutions (Marsh et al., 2008), or trust in health delivery systems (Østergaard, 2015; Tibbels, 2015). There is limited literature examining the impacts of research on trust between community volunteers and between these volunteers and the communities they come from. In this respect, our study contributes an additional perspective on the effects of community engagement on trust. We show explicitly how trust is intrinsically located within the social relations surrounding participants and delivery agents in community-based interventions. We also demonstrate how the nuances in changing practice in response to the need to achieve research outcomes impact on the fluidity of trust across different groups, particularly how the initial trust in CC roles reinforced through CR roles at the beginning of the intervention were lost when the same CRs were required to monitor the integrity of the CCs once trust had been established in the CC role. Thus trust for CCs began with distrust from the community, developed into trust in confidentiality through the support of the CRs and in parallel trust that was originally inherent in CCs due to their representative role towards the community was gradually eroded into distrust both through their role as key informants reporting death and finally through their role as informants or “watchdogs” on the CCs themselves. This placed the CRs in a challenging social position that eventually extended beyond the duration of the intervention, in some cases changing the very social relationships on which their selection as CRs had been based.

This project was relatively small in scope and whilst the participatory workshop and focus groups generated many interesting ideas we were only able to conduct a small number of interviews. Additional interviews could have provided further perspectives and helped to confirm findings. In addition, some issues were primarily reported by CCs and CRs, and might have benefited from further exploration with community members and the broader research team to understand their views.

The study was conducted one year after the trial finished. Ideally, it would have been helpful to conduct this study concurrently with the trial to examine changes in trust as they occurred *in situ* and to explore changing dynamics that reflected the changing dynamics of intervention design. This would have reduced participants’ reliance on recall and facilitated the ability of the researchers to address issues as they arose in order to reduce both short and long-term impacts on social relations within the study communities.

## Conclusions

Our study shows how multiple roles and the urgency to answer research questions can place pressure on study volunteers and create relations of distrust within communities. Studies need to carefully consider the impact of the roles assigned to community volunteers and the impact of altering defined roles described during recruitment in the course of study implementation. Changing the pre-defined roles of community volunteers recruited through an engaged, participatory process needs to reflect the engagement aim in itself by involving the community in these redefinitions, ensuring they are informed of these changes and sensitized to their purpose. Assessment of both feasibility and appropriateness of recruitment targets, and whether these roles and targets might have implications for social relationships and the wellbeing of study volunteers beyond the life of the trial or research is important at the formative phase of any community-based research. Negotiating social relationships within communities can be challenging, especially if community members develop new social roles through informal recruitment within an intervention trial. It is important to recognize the long-term impacts of community-based interventions that extend beyond the duration of the research and to take these into account when engaging communities actively in intervention activities, additionally ensuring that impact of such engagement activities is monitored and fed back to research teams in a timely feedback system to promote responsive and ethical research engagement.
